# Variations of wheat (*Triticum aestivum* L.) chromosomes caused by the 5A chromosomes with complex cytological structure

**DOI:** 10.3389/fpls.2022.992934

**Published:** 2022-08-29

**Authors:** Yang Zou, Jie Luo, Zongxiang Tang, Shulan Fu

**Affiliations:** ^1^College of Agronomy, Sichuan Agricultural University, Chengdu, China; ^2^Provincial Key Laboratory for Plant Genetics and Breeding, Chengdu, China

**Keywords:** wheat, 5A chromosome, meiotic recombination, chromosomal variation, tandem repeats

## Abstract

To study the effects of structural alterations of chromosomes caused by tandem repeats on the meiotic recombination, the wheat (*Triticum aestivum* L.) 5A chromosomes with different structure from ten wheat cultivars were used to investigate their meiotic recombination using non-denaturing fluorescence *in situ* hybridization (ND-FISH) technology. Fifteen cross combinations were carried out and they were divided into seven F_1_ categories. The structural difference between the intercalary regions of the long arms of the two 5A chromosomes (5AL) in the F_1_ categories III, VI, and VII was greater than that in the categories I and II, subsequently, the recombination frequencies in the distal regions of the 5AL arm in the progenies from the three categories were significantly lower than that from the categories I and II. For the two 5A chromosomes in the F_1_ categories VI and VII, the structural differences in the distal regions of both of the two arms were greater than that in the categories IV and V. So, the recombination frequencies in the intercalary region of the 5AL arm in the progeny from the categories IV and V were higher than that in the progeny from the categories VI and VII. The breakage of 5A chromosome together with the 5A translocations and the breakage of some other chromosomes were observed in the progeny from the F_1_ categories V, VI, and VII. These chromosomal variations were not observed in the progenies from the other four F_1_ categories. In conclusion, the smaller structural difference between the 5A chromosomes in distal regions of the two arms resulted in a higher recombination frequency in interstitial region and vice versa. The 5A chromosome with complex cytological structure can be used to induce genetic variations of wheat genome.

## Introduction

Wheat (*Triticum aestivum* L.) is rich in tandem repeats (Komuro et al., 2013; Tang et al., 2018; Lang et al., 2019; Zhu et al., 2021). These tandem repeats reflect the complex cytological structure of wheat chromosomes (Jiang et al., 2017; Huang et al., 2018; Guo et al., 2019; Hu et al., 2022). It is well known that there is a close relationship between the condensation of chromatin and tandem repeats (Gilbert et al., 2004; Ribeiro et al., 2017; Zou et al., 2021). Aggregation of several tandemly repeated clusters into a chromosomal segment resulted in more condensation of metaphase chromosome (Zou et al., 2021). It has been reported that highly condensed heterochromatin regions inhibit the initiation of crossover recombination and the acquisition of repair proteins (Brachet et al., 2012; Jacob et al., 2014; Underwood and Choi, 2019). These studies suggest that the degree of chromatin condensation and its spatial structure can affect the meiotic recombination. Repeated DNA sequences provide a unique higher-order structure of chromatin, and different chromatin states may affect the meiotic crossover recombination. It was reported that meiotic recombination might be affected by the constitution of tandem repeats (Zou et al., 2021). Whereas, effects of structural variations caused by tandem repeats on meiotic recombination are still largely unknown.

During meiosis, chromosomal recombination starts from programmed DNA double-strand breaks (DSBs), and a small amount of DSBs result in crossovers (COs) (Keeney et al., 1997; Lam and Keeney, 2014; Pazhayam et al., 2021). The smooth progress of the replication fork is an important link in the DSB repair process. Some secondary structures formed by repetitive sequences on the chromosome will hinder the replication fork process, thereby affecting the DSB repairing, and may further affect the meiotic recombination between homologous chromosomes (Zhang and Freudenreich, 2007; Brachet et al., 2012; Gadgil et al., 2017). Therefore, the high-level structure of chromosomes determined by repetitive sequences is closely related to the chromosome breakage, rearrangement and translocation (Molnár et al., 2011; Vader et al., 2011; Gadgil et al., 2017). Although some high-level structures formed by repetitive sequences have been found to affect chromosomal alterations, the more direct and intuitive evidence are still lack.

Wheat chromosome is a good material for studying the effect of tandem repeat composition and chromatin structure on meiotic recombination. Rich structural polymorphisms of 5A chromosomes in common wheat were reported (Hu et al., 2022). In this study, the meiotic recombination between 5A chromosomes was investigated, the chromosomal breakage and non-homologous recombination occurred on 5A chromosomes with complex cytological structure were observed, and this case was discussed.

## Materials and methods

### Plant materials

Ten wheat cultivars Chuanyu 17 (CY17), Mianyang 26 (MY26), Chuanmai 39 (CM39), Chuanmai 61 (CM61), Chuanmai 90 (CM90), Chuanmai 91 (CM91), 10jian236, CD012J41, Kechengmai 2 (KCM2), and Chuanshuangmai 1 (CSM1) were used as parents for hybridization. A total of 15 hybrid combinations, namely CM90 × CM61, MY26 × 10jian236, MY26 × CM61, CD012J41 × CM91, CD012J41 × 10jian236, CM39 × CM61, KCM2 × MY26, KCM2 × CY17, KCM2 × CM90, KCM2 × 10jian236, KCM2 × CM61, KCM2 × CM91, CSM1 × 10jian236, CSM1 × CM61, and CSM1 × CM91 were obtained. According to the FISH signal patterns of 5A chromosomes, the F_1_ plants were divided into seven categories I, II, III, IV, V, VI, and VII. The F_1_ generation was then selfed, and finally 15 F_2_ populations were obtained. A total of 2,068 grains from the F_2_ populations were analyzed.

### Non-denaturing fluorescence *in situ* hybridization

Germination of wheat seeds, pretreatment of root tips, and preparation of metaphase chromosomes were performed as described by Han et al. (2006). The procedure of ND-FISH was performed as described by Fu et al. (2015). The oligo probes Oligo-pSc119.2-1, Oligo-pTa535-1 (Tang et al., 2014), Oligo-713, Oligo-275.1 (Tang et al., 2016), and Oligo-18 (Tang et al., 2014) were used for ND-FISH analysis. The signals of the Oligo-275.1 and Oligo-18 probes are overlapped and represented as Oligo-18/Oligo-275.1. An epifluorescence microscope BX51 (Olympus Corporation, Tokyo, Japan) with cellSens Dimension software (Olympus Corporation, Tokyo, Japan) was used to capture images. FISH signal pattern was drawn using Adobe Photoshop CS 6.0.

### Statistics and analysis of data

The recombinant 5A chromosomes were judged according to the signal patterns of the oligo probes used in this study on 5A chromosomes. The recombinant chromosomes observed in the F_2_ populations of the seven F_1_ categories (I, II, III, IV, V, VI, and VII) were named as following: Single recombination was named Rec1-n, Rec2-n, Rec3-n, Rec4-n, Rec5-n Rec6-n, and Rec7-n, and so on. Double recombination were named DRec1-n, DRec2-n, DRec3-n, DRec4-n, DRec5-n, DRec6-n, and DRec7-n, and so on. Triple recombination was named TRec1-n, TRec2-n, TRec3-n, TRec4-n, TRec5-n, TRec6-n, and TRec7-n, and so on. The number of each type of 5A chromosome in F_2_ generation plants was counted.

Frequency of 5A chromosome breakage = number of broken 5A chromosomes/total number of 5A chromosomes from each hybrid combination × 100%. Frequency of 5A chromosome translocations = number of translocated 5A chromosomes/total number of 5A chromosomes from each hybrid combination × 100%. Recombination frequency in each interval = number of 5A chromosome from recombination in each interval/total number of 5A chromosomes derived from the corresponding F_1_ category × 100%. Chi-square test and *t*-test were used to determine significant differences in recombination frequency and chromosomal alteration, respectively. Graphing were performed using GraphPad Prism (version 8.0).

## Results

### Fluorescence *in situ* hybridization patterns of 5A chromosomes and F_1_ categories

**FIGURE 1:**
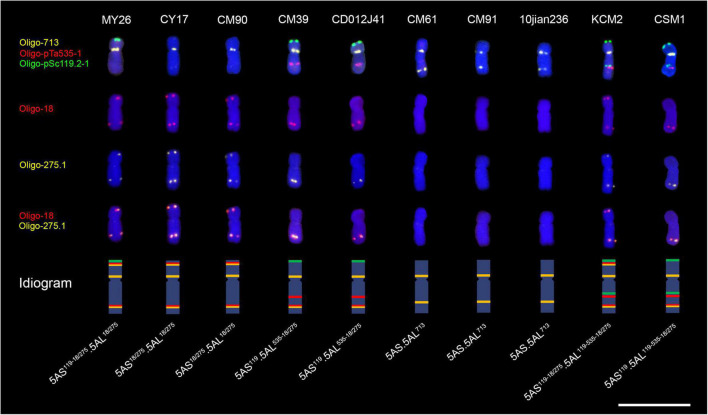
Signal patterns of five oligo probes on the root-tip metaphase 5A chromosomes of ten wheat cultivars. The ideogram of each chromosome is shown. Scale bar, 20 μm.

5A chromosomes in common wheat can be identified according to the signal patterns of oligo probes Oligo-pSc119.2-1, Oligo-pTa535-1, Oligo-713, Oligo-275.1, and Oligo-18 (Tang et al., 2014, 2016; Zou et al., 2021). In this study, the different structure of 5A chromosomes of ten wheat cultivars was displayed by the five oligo probes (Figure 1). Cultivars MY26, CM39, CD012J41, KCM2, and CSM1 contained Oligo-pSc119.2-1 signals in the telomeric region of the short arms of 5A chromosomes (5AS) (Figure 1). KCM2 and CSM1 contained Oligo-pSc119.2-1 signals in the intercalary regions of the long arms of 5A chromosomes (5AL) (Figure 1). The intercalary regions of 5AL arms of CM39, CD012J41, KCM2, and CSM1 carried Oligo-pTa535-1 signals. The Oligo-713 signals appeared in the subtelomeric region of 5AL arms of CM61, CM91, and 10jian236, and in the pericentromeric regions of all the 5AL arms investigated in this study (Figure 1). The subtelomeric regions of 5AS and 5AL arms of CY17, MY26, CM90, and KCM2 carried both Oligo-18 and Oligo-275.1 signals and the two kinds of signals overlapped with each other, and the overlapped Oligo-18 and Oligo-275.1 signals appeared in the subtelomeric regions of 5AL arms of CM39, CD012J41, and CSM1 (Figure 1). Based on signal patterns of these probes, the 5A chromosomes in the ten wheat cultivars were named as that listed in Table 1. The ideograms of different types of 5A chromosomes were also displayed (Figure 1).

**TABLE 1:** The name of the 5A chromosomes in the ten wheat cultivars.

Wheat cultivar	Name of 5A chromosome
MY26	5AS^119–18/275^.5AL^18/275^
CY17 and CM90	5AS^18/275^.5AL^18/275^
CM39 and CD012J41	5AS^119^.5AL^535–18/275^
CM61, CM91 and 10jian236	5AS.5AL^713^
KCM2	5AS^119–18/275^.5AL^119–535–18/275^
CSM1	5AS^119^.5AL^119–535–18/275^

**FIGURE 2:**
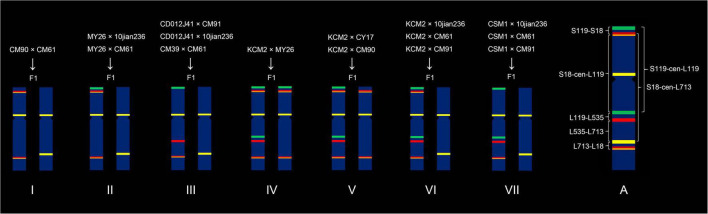
Seven categories of combination of 5A chromosomes in F_1_ generation plants. “F1” indicates F_1_ generation. (A) Scheme of recombination intervals.

A total of 15 hybrid combinations were carried out using the ten wheat cultivars, and these F_1_ plants were divided into seven categories according to their combinations of 5A chromosomes (Figure 2 and Supplementary Figure 1). The categories I, II, III, VI, and VII contained chromosomes 5AS^18/275^.5AL^18/275^, 5AS^119–18/275^.5AL^18/275^, 5AS^119^.5AL^535–18/275^, 5AS^119–18/275^.5AL^119–535–18/275^, and 5AS^119^.5AL^119–535–18/275^, respectively, and they all contained chromosome 5AS.5AL^713^ (Figure 2 and Supplementary Figure 1). Chromosomes 5AS^119–18/275^.5AL^18/275^ and 5AS^18/275^.5AL^18/275^ existed in the categories IV and V, respectively, and both of them contained 5AS^119–18/275^.5AL^119–535–18/275^ chromosome (Figure 2 and Supplementary Figure 1).

The 5A chromosomes in the 15 F_2_ populations were analyzed using ND-FISH, and the type of recombinant 5A chromosomes were investigated. Seven recombination intervals can be observed in these 5A chromosomes (Figure 2 and Table 2). The S119-S18 interval located between the signal sites of Oligo-pSc119.2-1 and Oligo-18/Oligo-275.1 on 5AS arm (Figure 2 and Table 2). The S119-cen-L119 interval was from the signal site of the Oligo-pSc119.2-1 on 5AS arm to that of Oligo-pSc119.2-1 on 5AL arm (Figure 2 and Table 2). The S18-cen-L713 interval was from the signal site of the Oligo-18/Oligo-257.1 on 5AS arm to that of Oligo-713 on 5AL arm (Figure 2 and Table 2). The S18-cen-L119 interval was from the Oligo-18 signal site on 5AS arm to the Oligo-pSc119.2-1 signal site on 5AL arm (Figure 2 and Table 2). All the three intervals were across the centromere. There are three recombination intervals on 5AL arm and they are the L119-L535 interval between Oligo-pSc119.2-1 and Oligo-pTa535-1 signal sites, the L535-L713 interval between Oligo-pTa535-1 and Oligo-713 signal sites, and the L713-L18 interval between Oligo-713 and Oligo-18 signal sites (Figure 2 and Table 2).

**TABLE 2:** Seven recombination intervals on 5A chromosome.

Recombination interval	S119-S18 interval	S119-cen-L119 interval	S18-cen-L713 interval	S18-cen-L119 interval	L119-L535 interval	L535-L713 interval	L713-L18 interval
Regions	Oligo-pSc119.2-1 to Oligo-18 on 5AS arm	Oligo-pSc119.2-1 of 5AS to Oligo-pSc119.2-1 of 5AL arm	Oligo-18 of 5AS to Oligo-713 of 5AL arm	Oligo-18 of 5AS to Oligo-pSc119.2-1 of 5AL arm	Oligo-pSc119.2-1 to Oligo-pTa535-1 on 5AL arm	Oligo-pTa535-1 to Oligo-713 on 5AL arm	Oligo-713 to Oligo-18 on 5AL arm
							

### Recombination of 5A chromosomes in F_2_ generations derived from F_1_ categories I, II, and III

Our original purpose was to investigate the recombination occurred on 5AL arm. The oligo probe Oligo-pSc119.2-1 was not used to analyze the 5A chromosomes in F_2_ generations derived from F_1_ categories I, II and III, because their 5AL arms did not contain pSc119.2 tandem repeats (Figure 2). A total of 812 5A chromosomes from 406 seeds of the F_2_ generations from the F_1_ categories I and II were analyzed. A total of 423 recombinant 5A chromosomes including four kinds of single recombinants and two kinds of double recombinants were observed (Supplementary Table 1 and Supplementary Figure 2). These recombinant 5A chromosomes were resulted from the recombination in S18-cen-L713 interval, L713-L18 interval and in both the two intervals (Supplementary Table 1 and Supplementary Figure 2). Eight hundred and four 5A chromosomes from 402 seeds of the F_2_ generation from the F_1_ category III were analyzed. There were also four kinds of single recombinant 5A chromosomes and two kinds of double recombinant 5A chromosomes (Supplementary Table 1 and Supplementary Figure 3). The 317 recombinant 5A chromosomes were resulted from the recombination in L535-L713 interval, L713-L18 interval, and in both the two intervals (Supplementary Table 1 and Supplementary Figure 3). In the F_2_ plants from the F_1_ categories I, II, and III, no chromosomal breakage and non-homologous recombination involved in 5A chromosomes were observed.

### Recombination of 5A chromosomes in the F_2_ generations derived from F_1_ categories IV–VII

Compared with F_1_ categories I, II, and III, the cytological structure of 5A chromosomes in F_1_ categories IV, V, VI, and VII was more complex (Figures 1, 2 and Supplementary Figure 1). A total of 2,521 5A chromosomes in the F_2_ generations derived from the F_1_ categories IV, V, VI, and VII were analyzed. In the F_2_ plants (292 5A chromosomes) from the F_1_ category IV, only two kinds of recombinant 5A chromosomes (Rec4-1 and Rec4-2) were observed, and the recombination occurred in the L119-L535 interval (Supplementary Table 1 and Supplementary Figure 4). In the F_2_ generation plants (586 5A chromosomes) from the F_1_ category V, the recombinant 5A chromosomes Rec5-1 and Rec5-2 were derived from the recombination in the S119-cen-L119 interval (Supplementary Table 1 and Supplementary Figure 4). The recombination in the L119-L535 interval produced the recombinant 5A chromosomes Rec5-3 and Rec5-4 (Supplementary Table 1 and Supplementary Figure 4). The recombinant 5A chromosomes DRec5-1 and DRec5-2 were derived from the recombination in both the S119-cen-L119 and the L119-L535 intervals (Supplementary Table 1 and Supplementary Figure 4). The broken chromosomes and non-homologous recombination chromosomes were not observed in the F_2_ generation from the F_1_ category IV.

**FIGURE 3:**
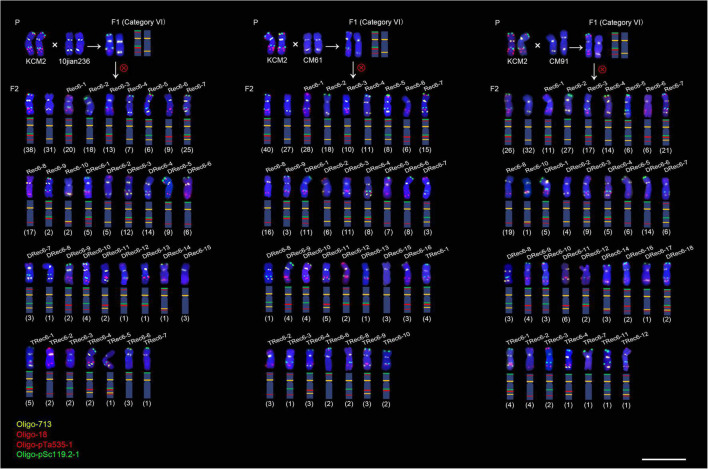
Fluorescence *in situ* hybridization signal patterns of the 5A chromosomes in progeny of three hybrid combinations of category VI. “P” indicates parental plants. “F1” indicates F_1_ generation. “F2” indicates F_2_ generation. Rec6-n, DRec6-n and TRec6-n represent the single recombination, double recombination and triple recombination, respectively. The numbers in parentheses indicate the number of each type of 5A chromosome. The schematic representation of each chromosome is shown. Scale bar, 20 μm.

In the F_2_ generation plants (833 5A chromosomes) from the F_1_ category VI, a total of 40 types of recombinant 5A chromosomes including 10 kinds of single recombinants (Rec6-1 to Rec6-10), 18 kinds of double recombinants (DRec6-1 to DRec6-18), and 12 kinds of triple recombinants (TRec6-1 to TRec6-12) were observed (Figure 3 and Supplementary Table 1). In the F_2_ generation plants (810 5A chromosomes) from the F_1_ category VII, 22 types of recombinant 5A chromosomes including 8 kinds of single recombinants (Rec7-1 to Rec7-8), 10 kinds of double recombinants (DRec7-1 to DRec7-10), and 4 kinds of triple recombinants (TRec7-1 to TRec7-4) were observed (Supplementary Table 1 and Supplementary Figure 5). The recombinant 5A chromosomes derived from both the VI and VII F_1_ categories were resulted from the recombination in the S119-S18, S119-cen-L119, S18-cen-L119, L119-L535, L535-L713, and the L713-L18 intervals (Figure 3 and Supplementary Table 1 and Supplementary Figure 5).

### Recombination frequency of 5A chromosomes

**FIGURE 4:**
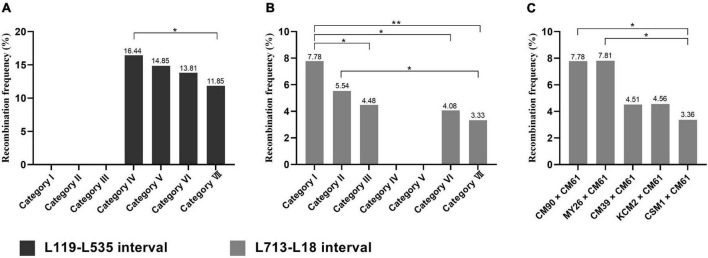
Recombination frequency of 5A chromosome in L119-L535 and L713-L18 intervals. **(A)** Recombination frequency of 5A chromosome in L119-L535 interval. **(B,C)** Recombination frequency of 5A chromosome in L713-L18 interval. *p < 0.05, **p < 0.01 (Chi-square test).

The recombination frequency for each interval that can be determined in the progeny from the seven F_1_ categories was listed in Table 3. It can be noted that recombination interval S119-cen-L199 contained the two intervals S119-S18 and S18-cen-L119 (Table 2 and Figure 2). Likewise, the recombination interval S18-cen-L713 contained the three intervals S18-cen-119, L119-L535, and L535-L713 (Table 2 and Figure 2). The recombination frequency in the S119-cen-L199 interval in the progeny from the F_1_ categories V and VII reflected the recombination of two intervals, and that in the S18-cen-L713 interval in the progeny from the F_1_ categories I and II reflected the recombination of three intervals (Table 3). Therefore, only the frequencies of the recombination occurred in the L119-L535 interval among the progeny of F_1_ categories IV, V, VI, and VII (Figure 4A), in the L713-L18 interval among the progeny of F_1_ categories I, II, III, VI, and VII (Figure 4B), and in the L713-L18 interval among the progeny from the five hybrid combinations involved in CM61 (Figure 4C) were compared. It can be noted that the recombination frequency in the L119-L535 interval in the progeny of F_1_ category IV was the highest (16.44%), and it was significantly higher than that in the progeny of the F_1_ category VII (11.85%) (*P* < 0.05) (Figure 4A). For the L713-L18 interval, the recombination frequency in the progeny of F_1_ category I was the highest (7.78%), and it was the lowest (3.33%) in the progeny of the F_1_ category VII (Figure 4B). Great differences were observed among the progeny from F_1_ categories I, II, III, VI and VII (*P* < 0.01) (Figure 4B). Additionally, the recombination frequencies in the L713-L18 interval in the progeny from CM90 × CM61 and MY26 × CM61 (categories I and II) were significantly higher than that in the progeny from CM39 × CM61, KCM2 × CM61, and CSM1 × CM61 (categories III, VI, and VII) (*P* < 0.05) (Figure 4C).

**TABLE 3:** Recombination frequencies of 5A chromosome in each intervals in F_2_ population from seven F_1_ categories (I–VII)*.

Interval Category	S119-S18 interval (%)	S18-cen-L119 interval (%)	L119-L535 interval (%)	L535-L713 interval (%)	L713-L18 interval (%)
I	—	49.26			7.78
II	—	47.60			5.54
III	—	—	—	36.69	4.48
IV	—	—	16.44	—	—
V	41.81		14.85	—	—
VI	34.21	24.73	13.81	36.01	4.08
VII	49.01		11.85	42.59	3.33

*The S119-cen-L119 interval contains the S119-S18 and S18-cen-L119 intervals. The S18-cen-L713 interval contains the S18-cen-L119, L119-L535, and L535-L713 intervals. “—” Indicates data not available.

### Breakage and non-homologous recombination of 5A chromosomes

**FIGURE 5:**
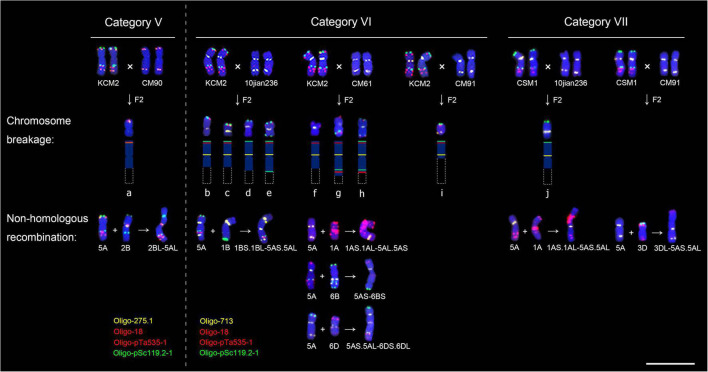
Fluorescence *in situ* hybridization signal patterns of the broken 5A chromosomes and non-homologous recombination chromosomes. “ × “ indicates hybridization. “F2” indicates F_2_ generation. “ + “ indicates translocation. Dotted boxes indicates missing parts of chromosomes. Scale bar, 20 μm.

**FIGURE 6:**
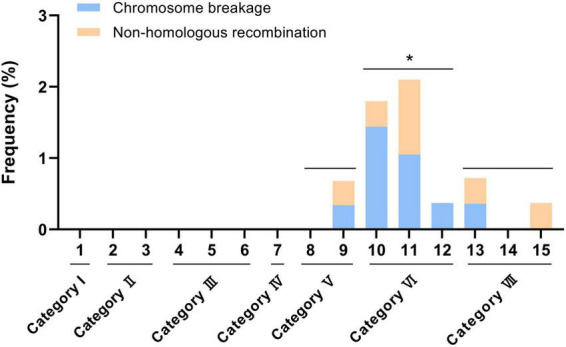
Frequency of broken and non-homologous recombination 5A chromosomes. Numbers in horizontal axis indicate the 15 hybrid combination. *p < 0.05 (*t*-test).

In this study, a total of ten broken 5A chromosomes and seven non-homologous recombination chromosomes were found in the F_2_ generations from the F_1_ categories V, VI, and VII (Figure 5 and Supplementary Table 2 and Supplementary Figures 6, 7). A broken 5A chromosome (a-5A^del^) and a 2BL-5AL translocation chromosome were found in two seeds from KCM2 × CM90 (category V), and the frequencies were 0.34% (1/292) and 0.34% (1/292), respectively (Figure 5 and Supplementary Figures 6A, 7A). Four broken 5A chromosomes (b-5A^del^, c-5A^del^, d-5A^del^, and e-5A^del^) and a 1BS.1BL-5AS.5AL translocation chromosome were found in five seeds from KCM2 × 10jian236 (category VI), and the frequencies were 1.44% (4/278) and 0.36% (1/278), respectively (Figure 5 and Supplementary Figures 6B–E, 7B). Three broken 5A chromosomes (f-5A^del^, g-5A^del^, and h-5A^del^) and three translocation chromosomes (1AS.1AL-5AL.5AS, 5AS-6BS, 5AS.5AL-6DS.6DL) were found in six seeds from KCM2 × CM61 (category VI), and the frequencies were 1.05% (3/285) and 1.05% (3/285), respectively (Figure 5 and Supplementary Figures 6F–H, 7C–E). A broken 5A chromosome (i-5A^del^) was found in one seed derived from KCM2 × CM91 (category VI) with a frequency of 0.37% (1/270) (Figure 5 and Supplementary Figure 6I). A broken 5A chromosome (j-5A^del^) and a 1AS.1AL-5AS.5AL translocation chromosome were found in two seeds derived from CSM1 × 10jian236 (category VII), and the frequencies were 0.36% (1/274) and 0.36% (1/274), respectively (Figure 5 and Supplementary Figures 6J, 7F). A 3DL-5AS.5AL translocation chromosome was found in one seed derived from CSM1 × CM91 (category VII) with a frequency of 0.37% (1/268) (Figure 5 and Supplementary Figure 7G). Among them, the frequency of broken 5A chromosomes in the progeny from the F_1_ category VI was significantly higher than that in the progeny from the F_1_ categories V and VII (*P* < 0.05) (Figure 6). In a seed that contained h-5A^del^ broken chromosome, two intact 5A chromosomes were also observed (Supplementary Figure 6H). This indicates that the 5A chromosomes in this seed experienced an abnormal meiosis.

According to the signal distribution of oligo probes on 5A chromosomes, it can be determined that the broken 5A chromosomes a-5A^del^, b-5A^del^, c-5A^del^, e-5A^del^, f-5A^del^, g-5A^del^, h-5A^del^, and i-5A^del^ were derived from the breakage in the chromosome 5AS^119–18/275^.5AL^119–535–18/275^, the broken 5A chromosome j-5A^del^ was from the breakage of 5AS^119^.5AL^119–535–18/275^, and the broken 5A chromosome d-5A^del^ was from chromosomes 5AS^119–18/275^.5AL^119–535–18/275^ or 5AS.5AL^713^ (Figure 5). All the breakage occurred in the 5AL arms. The breakpoints in the chromosomes a-5A^del^, b-5A^del^, c-5A^del^, d-5A^del^, f-5A^del^, and j-5A^del^ were in the intercalary regions between the centromere and the Oligo-pSc119.2-1 signal site, the breakpoint in e-5A^del^ was between the signal sites of Oligo-pSc119.2-1 and Oligo-pTa535-1, the breakage near the centromere resulted in the i-5A^del^, and the breakage between the signal sites of Oligo-pTa535-1 and Oligo-18/Oligo-275.1 resulted in the chromosomes g-5A^del^ and h-5A^del^ (Figure 5).

### Breakage of other chromosomes derived from the F_1_ categories VI and VII

**FIGURE 7:**
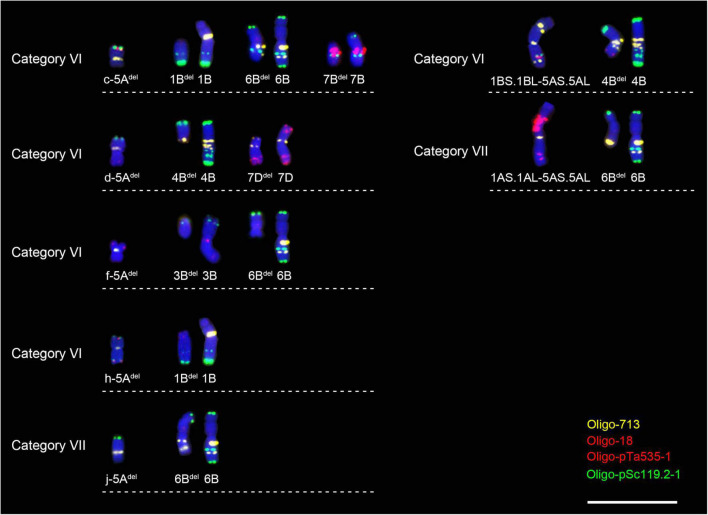
Other broken chromosomes in the cells with broken 5A chromosome or 5A translocation. Scale bar, 20 μm.

In some cells with broken 5A chromosomes or 5A translocations, the breakage of other chromosomes was also observed. In the cell with c-5A^del^, the broken 1B, 6B, and 7B chromosomes (1B*^del^*, 6B^del^, and 7B^del^) were found (Figure 7 and Supplementary Figure 6C). In the cell with d-5A^del^, the broken 4B and 7D chromosomes (4B^del^ and 7D^del^) were found (Figure 7 and Supplementary Figure 6D). In the cell containing f-5A^del^, the broken 3B and 6B chromosomes (3B^del^ and 6B^del^) were found (Figure 7 and Supplementary Figure 6F). There were two broken and a intact 1B chromosomes in the cell with h-5A^del^ (Figure 7 and Supplementary Figure 6H). Two different broken 6B chromosomes (6B^del^) was observed in the cell with j-5A^del^ and the cell with 1AS.1AL-5AS.5AL translocation chromosome, respectively (Figure 7 and Supplementary Figures 6J, 7F). In the cell with 1BS.1BL-5AS.5AL translocation chromosome, the broken 4B (4B^del^) were found (Figure 7 and Supplementary Figure 7B). However, in all the other cells without broken 5A chromosomes or 5A translocations, the chromosome breakage and translocation were not found. These phenomena suggested that the structural variations of 5A chromosome can affect the stability of other chromosomes. In addition, the stable generation transmission of these broken 5A chromosomes and other broken chromosomes was observed.

## Discussion

### The effects of compositional differences in tandem repeats on recombination of 5A chromosomes

The 5A chromosomes used in this study have abundant FISH signal patterns. It can be noted that the structural difference between the 5A chromosomes in category IV was observed only in the region of L119-L535 interval (Figure 2). In categories V, VI, and VII, the structural differences between the 5A chromosomes were observed in the telomeric regions on 5AS arms and in the subtelomeric regions on 5AL arms (Figure 2). Therefore, the structural difference between the 5A chromosomes in category IV was smaller than that in categories V, VI, and VII (Figure 2), and this was in accordance with that the recombination frequency in the L119-L535 interval in the category IV was higher than that in the categories V, VI, and VII (Figure 4A). The similar phenomenon was observed from the recombination frequencies in the L713-L18 interval among the progeny of F_1_ categories I, II, III, VI, and VII (Figures 2, 4B). That is, the smaller structural difference between the 5A chromosomes in the L119-L535 interval resulted in a higher recombination frequency in the L713-L18 interval (Figures 2, 4B). The higher recombination frequency in the L713-L18 interval in the progeny from CM90 × CM61 and MY26 × CM61 than that in the progeny from CM39 × CM61, KCM2 × CM61, and CSM1 × CM61 also supported this conclusion. Although the recombination frequencies of wheat chromosomes were affected by many factors including transposon elements (Darrier et al., 2017), sequence insertions/deletions (Saintenac et al., 2011) and single nucleotide polymorphisms (SNPs) (Jordan et al., 2018), etc., the different composition of tandem repeats in chromosomes might be one of the factors affecting meiotic recombination, because the structural variations in 5A chromosomes are consistence with the different recombination frequencies. The results obtained in this study agree with that reported by Zou et al. (2021). During meiosis, a chromatin remodeling in chromosomes are needed for the homologous chromosome recognition and pairing (Prieto et al., 2004; Colas et al., 2008). The study on the meiosis of wheat-rye 1BL.1RS translocation chromosomes indicated that the chromatin remodeling was not observed during the bouquet formation when the structure of the subtelomeric regions of the 1RS arms was different, and this affected the subsequent recombination (Colas et al., 2008). However, the conformation changes in the subtelomeric regions of the 1RS arms occurred when their structure is identical (Colas et al., 2008). So, the conformation changes of the 5A chromosomes with significant structural difference might be different and subsequently their meiotic pairing was hindered, and this resulted in the lower recombination.

### Possible reason leading the breakage of 5A chromosome and non-homologous recombination

Among the F_2_ populations, the breakage and the non-homologous recombination involved in 5A chromosomes were only observed in the progeny derived from the F_1_ categories V, VI, and VII, (Figures 5, 6). In all the three kinds of F_1_ categories, the intercalary region of one of the 5AL arm contained two tandem repeats pSc119.2 and pTa-535, and the other one did not contain them (Figures 2, 5). Therefore, the regions of the L119-L535 interval in these 5AL arms displayed obvious structural difference (Figure 2). This structural difference was not observed among the 5AL arms in F_1_ categories I, II, and III (Figure 2), accordingly, no chromosome breakage and translocation were observed in the progeny of all the three F_1_ categories. Therefore, it was presumed that the structural difference caused by tandem repeats might be an important factor resulting in the breakage and non-homologous recombination of 5A chromosomes.

Most of the breakpoints in the 5AL arms were located around the L119-L535 interval (Figure 5). Studies have reported that chromosomal rearrangements frequently occur in the border regions of heterochromatin and euchromatin (Badaeva et al., 2007; Raskina et al., 2008). The more copies of repeat sequence may increase DSB formation (Bose et al., 2014). Therefore, the DSB frequency might increase in the regions around the L119-L535 interval because of the clustering of tandem repeats pSc119.2 and pTa-535. At the beginning of meiosis recombination, the chromatin in the interstitial and pericentromeric regions maintain a highly condensed state, which then gradually opens, accompanied by assembly of the SC and recombination (Lenykó-Thegze et al., 2021). A close correlation existed between the SC formation and the meiosis recombination (Pyatnitskaya et al., 2019; Grey and de Massy, 2022). So, the assembly of the SC in the highly condensed regions enriched with tandem repeats might be blocked, which directly affects the subsequent recombination. Moreover, regions with highly condensation state also have poor chromatin accessibility, and this prevents the DSB repairing (Tock and Henderson, 2018; Aleksandrov et al., 2020). Our previous studies have found that the regions containing both pSc119.2 and pTa535 tandem repeats on the 5AL arms were more condensed than that without these two tandem repeats (Zou et al., 2021). It can be presumed that the DSB repairing in the regions around the L119-L535 interval might be hindered by the condensation state of this region, and the unsuccessful DSB repairing generated the breakage in 5A chromosomes. Additionally, the unsuccessful DSB repairing and homologous recombination might be also caused by the different chromatin remodeling between homologous chromosomes with different structure (Colas et al., 2008). The breakage and translocation involved in 5A chromosomes were not observed in F_1_ category IV, although one of the 5A chromosomes in this category contained both pSc119.2 and pTa-535. The similar structure of the telomeric and subtelomeric regions between the 5A chromosomes in the F_1_ category IV might induce their synchronized chromatin remodeling, subsequently might prompt their DSB repairing and recombination, and no breakage occurred. The frequencies of breakage and translocation in 5A chromosomes in the F_1_ category VI were significantly higher than that in the categories V and VII, and this was accordance with the greater difference between the 5A chromosomes in category VI than that in the categories V and VII. This case also indicated that the greater structural difference between 5A chromosomes, the more likely they were to break.

It was speculated that translocation hotspots may be located near heterochromatin regions, however, there is not enough evidence (Badaeva et al., 2007). The results in this study provided a direct evidence for that translocation can occur in the regions clustered with tandem repeats. Abnormal repair by homologous recombination (HR) can lead to chromosomal translocations during DSB repair (Pfeiffer et al., 2000; Rothkamm et al., 2003). It has been found that translocations between the genomes of *Aegilops* species often involve non-homologous chromosomes (Badaeva et al., 2002). In this study, the normal DSB repairing in 5A chromosomes were hindered, and the broken DNA double-strand might use the DNA strands from non-homologous chromosomes as template for DSB repairing, at low frequency, resulting in the translocations between 5A chromosome and its non-homologous chromosomes.

### Chromosome 5A may be involved in the regulation of chromosome pairing

In wheat, the *Ph1* gene is a regulator of normal chromosome pairing (Gyawali et al., 2019). Previous studies have found that the *Ph1* candidate gene (*C-Ph1*) has highly similar homologous genes on chromosomes 5A, 5B and 5D, namely *C-Ph1-5A*, *C-Ph1-5B*, and *C-Ph1-5D*. In the regulation of chromosome pairing, *C-Ph1-5B* plays a major role, *C-Ph1-5D* plays a partial role in early meiosis, and *C-Ph1-5A* has little function (Bhullar et al., 2014; Rawale et al., 2019). The *C-Ph1-5A* gene locus is located in the vicinity of 472Mb of 5AL (Zhu et al., 2021), and the L119-L535 interval is located in the 479Mb-527Mb region of 5AL (Zou et al., 2021), indicating that *C-Ph1-5A* locus is adjacent to the L119-L535 interval. In this study, in cells with broken and translocated 5A chromosomes, other chromosome breakage was also observed. Therefore, it was speculated that the *C-Ph1-5A* gene on chromosome 5A might be also involved in the control of meiosis in some special conditions. When the breakage and translocation occurred around the L119-L535 interval in 5A chromosome, the chromatin structure at the breakpoints will undergo significant changes, and this may affect the expression of *C-Ph1-5A* gene and subsequently change the regulation process of the entire *Ph1* system.

## Conclusion

The meiotic recombination between wheat 5A chromosomes was affected by their great structural difference. The smaller structural difference between the 5A chromosomes in the distal regions of the two arms resulted in a higher recombination frequency in the interstitial region and vice versa. The complex cytological structure of chromosomes caused by tandem repeats might prevent the normal DSB repairing, and subsequently might result in chromosomal breakage and no-homologous recombination. Additionally, the *Ph1* system might also be affected by the structural variations of 5A chromosomes. The 5A chromosome with complex cytological structure can be used to induce genetic variations in wheat genome.

## Data availability statement

The original contributions presented in the study are included in the article/Supplementary material, further inquiries can be directed to the corresponding authors.

## Author contribution

YZ and JL performed the experiments and analyzed the data. YZ wrote the draft manuscript. ZT and SF conceived and designed the study, analyzed the data, and edited the manuscript. All authors contributed to the article and approved the submitted version.

## Supplementary material

Supplementary Figure 1 Non-denaturing FISH (ND-FISH) analysis of the root-tip metaphase chromosomes of 15 F_1_ plants. The oligo probes Oligo-713 (green or yellow), Oligo-275.1 (yellow), Oligo-18 (red), Oligo-pTa535-1 (red), and Oligo-pSc119.2-1 (green) were used for ND-FISH analysis. The 15 F_1_ plants were divided into seven categories I, II, III, IV, V, VI, and VII. Scale bar, 20 μm.

Supplementary Figure 2 Fluorescence *in situ* hybridization signal patterns of 5A chromosomes in the F_2_ plants from the I and II F_1_ categories. “P” indicates parental plants. “F1” indicates F_1_ generation. “F2” indicates F_2_ generation. Numbers in parentheses indicate the number of corresponding 5A chromosome. The schematic representation of each 5A chromosome is shown. Scale bar, 20 μm.

Supplementary Figure 3 Fluorescence *in situ* hybridization signal patterns of 5A chromosomes in the F_2_ plants from the III F_1_ category. “P” indicates parental plants. “F1” indicates F_1_ generation. “F2” indicates F_2_ generation. Numbers in parentheses indicates the number of corresponding 5A chromosome. The schematic representation of each 5A chromosome is shown. Scale bar, 20 μm.

Supplementary Figure 4 Fluorescence *in situ* hybridization signal patterns of 5A chromosomes in the F_2_ plants from the IV and V F_1_ categories. “P” indicates parental plants. “F1” indicates F_1_ generation. “F2” indicates F_2_ generation. Numbers in parentheses indicates the number of corresponding 5A chromosome. The schematic representation of each 5A chromosome is shown. Scale bar, 20 μm.

Supplementary Figure 5 Fluorescence *in situ* hybridization signal patterns of the 5A chromosomes in the F_2_ plants from the three hybrid combinations of category VII. “P” indicates parental plants. “F1” indicates F_1_ generation. “F2” indicates F_2_ generation. Rec7-n, DRec7-n, and TRec7-n represent the single recombination, double recombination and triple recombination, respectively. The numbers in parentheses indicate the number of each type of 5A chromosome. The schematic representation of each chromosome is shown. Scale bar, 20 μm.

Supplementary Figure 6 Fluorescence *in situ* hybridization signal patterns of ten broken 5A chromosomes. The oligo probes Oligo-713 (yellow), Oligo-18 (red), Oligo-pTa535-1 (red), and Oligo-pSc119.2-1 (green) were used for ND-FISH analysis. Scale bar, 20 μm.

Supplementary Figure 7 Fluorescence *in situ* hybridization signal patterns of seven 5A non-homologous recombination chromosomes. The oligo probes Oligo-713 (yellow), Oligo-18 (red), Oligo-pTa535-1 (red), and Oligo-pSc119.2-1 (green) were used for ND-FISH analysis. Scale bar, 20 μm.

## References

[B1] Aleksandrov R. Hristova R. Stoynov S. Gospodinov A. (2020). The chromatin response to double-strand DNA breaks and their repair. *Cells* 9:1853. 10.3390/cells9081853 32784607 PMC7464352

[B2] Badaeva E. D. Amosova A. V. Muravenko O. V. Samatadze T. E. Chikida N. N. Zelenin A. V. (2002). Genome differentiation in *Aegilops*. 3. Evolution of the U-genome cluster. *Plant Syst. Evol.* 246 45–76. 10.1007/s006060200018

[B3] Badaeva E. D. Dedkova O. S. Gay G. Pukhalskyi V. A. Zelenin A. V. Bernard S. (2007). Chromosomal rearrangements in wheat: Their types and distribution. *Genome* 50 907–926. 10.1139/g07-072 18059554

[B4] Bhullar R. Nagarajan R. Bennypaul H. Sidhu G. K. Gill K. S. (2014). Silencing of a metaphase I-specific gene results in a phenotype similar to that of the Pairing homeologous 1 (Ph1) gene mutations. *Proc. Natl. Acad. Sci. U.S.A.* 111 14187–14192. 10.1073/pnas.1416241111 25232038 PMC4191769

[B5] Bose P. Hermetz K. E. Conneely K. N. Rudd M. K. (2014). Tandem repeats and G-rich sequences are enriched at human CNV breakpoints. *PLoS One* 9:e101607. 10.1371/journal.pone.0101607 24983241 PMC4090240

[B6] Brachet E. Sommermeyer V. Borde V. (2012). Interplay between modifications of chromatin and meiotic recombination hotspots. *Biol. Cell* 104 51–69. 10.1111/boc.201100113 22188336

[B7] Colas I. Shaw P. Prieto P. Wanous M. Spielmeyer W. Mago R. (2008). Effective chromosome pairing requires chromatin remodeling at the onset of meiosis. *Proc. Natl. Acad. Sci. U.S.A.* 105 6075–6080. 10.1073/pnas.0801521105 18417451 PMC2329686

[B8] Darrier B. Rimbert H. Balfourier F. Pingault L. Josselin A. A. Servin B. (2017). High-resolution mapping of crossover events in the hexaploid wheat genome suggests a universal recombination mechanism. *Genetics* 206 1373–1388. 10.1534/genetics.116.196014 28533438 PMC5500137

[B9] Fu S. Chen L. Wang Y. Li M. Yang Z. Qiu L. (2015). Oligonucleotide probes for ND-FISH analysis to identify rye and wheat chromosomes. *Sci. Rep.* 5:10552. 10.1038/srep10552 25994088 PMC4440213

[B10] Gadgil R. Barthelemy J. Lewis T. Leffak M. (2017). Replication stalling and DNA microsatellite instability. *Biophys Chem.* 225 38–48. 10.1016/j.bpc.2016.11.007 27914716 PMC5440219

[B11] Gilbert N. Boyle S. Fiegler H. Woodfine K. Carter N. P. Bickmore W. A. (2004). Chromatin architecture of the human genome: Gene-rich domains are enriched in open chromatin fibers. *Cell* 118 555–566. 10.1016/j.cell.2004.08.011 15339661

[B12] Grey C. de Massy B. (2022). Coupling crossover and synaptonemal complex in meiosis. *Genes Dev.* 36 4–6. 10.1101/gad.349286.121 35022326 PMC8763052

[B13] Guo J. Gao D. Gong W. Li H. Li J. Li G. (2019). Genetic diversity in common wheat lines revealed by fluorescence in situ hybridization. *Plant Syst. Evol.* 305 247–254. 10.1007/s00606-019-1567-y

[B14] Gyawali Y. Zhang W. Chao S. Xu S. Cai X. (2019). Delimitation of wheat ph1b deletion and development of ph1b-specific DNA markers. *Theor. Appl. Genet.* 132 195–204. 10.1007/s00122-018-3207-2 30343385

[B15] Han F. Lamb J. C. Birchler J. A. (2006). High frequency of centromere inactivation resulting in stable dicentric chromosomes of maize. *Proc. Natl. Acad. Sci. U.S.A.* 103 3238–3243. 10.1073/pnas.0509650103 16492777 PMC1413895

[B16] Hu Z. Luo J. Wan L. Luo J. Li Y. Fu S. (2022). Chromosomes polymorphisms of Sichuan wheat cultivars displayed by ND-FISH landmarks. *Cereal Res. Commun.* 50 253–262. 10.1007/s42976-021-00173-x

[B17] Huang X. Zhu M. Zhuang L. Zhang S. Wang J. Chen X. (2018). Structural chromosome rearrangements and polymorphisms identified in Chinese wheat cultivars by high-resolution multiplex oligonucleotide FISH. *Theor. Appl. Genet.* 131 1967–1986. 10.1007/s00122-018-3126-2 29947816

[B18] Jacob Y. Bergamin E. Donoghue M. T. Mongeon V. LeBlanc C. Voigt P. (2014). Selective methylation of histone H3 variant H3.1 regulates heterochromatin replication. *Science* 343 1249–1253. 10.1126/science.1248357 24626927 PMC4049228

[B19] Jiang M. Xaio Z. Fu S. Tang Z. (2017). FISH karyotype of 85 common wheat cultivars/lines displayed by ND-FISH using oligonucleotide probes. *Cereal Res. Commun.* 45 549–563. 10.1556/0806.45.2017.049

[B20] Jordan K. W. Wang S. He F. Chao S. Lun Y. Paux E. (2018). The genetic architecture of genome-wide recombination rate variation in allopolyploid wheat revealed by nested association mapping. *Plant J.* 95 1039–1054. 10.1111/tpj.14009 29952048 PMC6174997

[B21] Keeney S. Giroux C. N. Kleckner N. (1997). Meiosis-specific DNA double-strand breaks are catalyzed by Spo11, a member of a widely conserved protein family. *Cell* 88 375–384. 10.1016/s0092-8674(00)81876-0 9039264

[B22] Komuro S. Endo R. Shikata K. Kato A. Scoles G. (2013). Genomic and chromosomal distribution patterns of various prepeated DNA sequences in wheat revealed by a fluorescence in situ hybridization procedure. *Genome* 56 131–137. 10.1139/gen-2013-0003 23659696

[B23] Lam I. Keeney S. (2014). Mechanism and regulation of meiotic recombination initiation. *Cold Spring Harb. Perspect. Biol.* 7:a016634. 10.1101/cshperspect.a016634 25324213 PMC4292169

[B24] Lang T. Li G. Wang H. Yu Z. Chen Q. Yang E. (2019). Physical location of tandem repeats in the wheat genome and application for chromosome identification. *Planta* 249 663–675. 10.1007/s00425-018-3033-4 30357506

[B25] Lenykó-Thegze A. Fábián A. Mihók E. Makai D. Cseh A. Sepsi A. (2021). Pericentromeric chromatin reorganisation follows the initiation of recombination and coincides with early events of synapsis in cereals. *Plant J.* 107 1585–1602. 10.1111/tpj.15391 34171148

[B26] Molnár I. Cifuentes M. Schneider A. Benavente E. Molnár-Láng M. (2011). Association between simple sequence repeat-rich chromosome regions and intergenomic translocation breakpoints in natural populations of allopolyploid wild wheats. *Ann. Bot.* 107 65–76. mcq215 10.1093/aob/21036694 PMC3002473

[B27] Pazhayam N. M. Turcotte C. A. Sekelsky J. (2021). Meiotic crossover patterning. *Front. Cell Dev. Biol.* 9:681123. 10.3389/fcell.2021.681123 34368131 PMC8344875

[B28] Pfeiffer P. Goedecke W. Obe G. (2000). Mechanisms of DNA double-strand break repair and their potential to induce chromosomal aberrations. *Mutagenesis* 15 289–302. 10.1093/mutage/15.4.289 10887207

[B29] Prieto P. Shaw S. Moore G. (2004). Homologue recognition during meiosis is associated with a change in chromatin conformation. *Nat. Cell Biol.* 6 906–908. 10.1038/ncb1168 15340450

[B30] Pyatnitskaya A. Borde V. De Muyt A. (2019). Crossing and zipping: Molecular duties of the ZMM proteins in meiosis. *Chromosoma* 128 181–198. 10.1007/s00412-019-00714-8 31236671

[B31] Raskina O. Barber J. C. Nevo E. Belyayev A. (2008). Repetitive DNA and chromosomal rearrangements: Speciation-related events in plant genomes. *Cytogenet. Genome. Res.* 120 351–357. 10.1159/000121084 18504364

[B32] Rawale K. S. Khan M. A. Gill K. S. (2019). The novel function of the Ph1 gene to differentiate homologs from homoeologs evolved in *Triticum turgidum* ssp. dicoccoides via a dramatic meiosis-specific increase in the expression of the 5B copy of the C-Ph1 gene. *Chromosoma* 128 561–570. 10.1007/s00412-019-00724-6 31494715

[B33] Ribeiro T. Marques A. Novák P. Schubert V. Vanzela A. L. Macas J. (2017). Centromeric and non-centromeric satellite DNA organisation differs in holocentric Rhynchospora species. *Chromosoma* 126 325–335. 10.1007/s00412-016-0616-3 27645892

[B34] Rothkamm K. Krüger I. Thompson L. H. Löbrich M. (2003). Pathways of DNA double-strand break repair during the mammalian cell cycle. *Mol. Cell. Biol.* 23 5706–5715. 10.1128/MCB.23.16.5706-5715.2003 12897142 PMC166351

[B35] Saintenac C. Faure S. Remay A. Choulet F. Ravel C. Paux E. (2011). Variation in crossover rates across a 3-Mb contig of bread wheat (*Triticum aestivum*) reveals the presence of a meiotic recombination hotspot. *Chromosoma* 120 185–198. 10.1007/s00412-010-0302-9 21161258

[B36] Tang S. Qiu L. Xiao Z. Fu S. Tang Z. (2016). New oligonucleotide probes for ND-FISH analysis to identify barley chromosomes and to investigate polymorphisms of wheat chromosomes. *Genes* 7:118. 10.3390/genes7120118 27929398 PMC5192494

[B37] Tang S. Tang Z. Qiu L. Yang Z. Li G. Lang T. (2018). Developing new oligo probes to distinguish specific chromosomal segments and the a, B, D genomes of wheat (*Triticum aestivum* L.) using ND-FISH. *Front. Plant Sci.* 9:1104. 10.3389/fpls.2018.01104 30093909 PMC6070686

[B38] Tang Z. Yang Z. Fu S. (2014). Oligonucleotides replacing the roles of repetitive sequences pAs1, pSc119.2, pTa-535, pTa71, CCS1, and pAWRC.1 for FISH analysis. *J. Appl. Genet.* 55 313–318. 10.1007/s13353-014-0215-z 24782110

[B39] Tock A. J. Henderson I. R. (2018). Hotspots for initiation of meiotic recombination. *Front. Genet.* 9:521. 10.3389/fgene.2018.00521 30467513 PMC6237102

[B40] Underwood C. J. Choi K. (2019). Heterogeneous transposable elements as silencers, enhancers and targets of meiotic recombination. *Chromosoma* 128 279–296. 10.1007/s00412-019-00718-4 31332531

[B41] Vader G. Blitzblau H. G. Tame M. A. Falk J. E. Curtin L. Hochwagen A. (2011). Protection of repetitive DNA borders from self-induced meiotic instability. *Nature* 477 115–119. 10.1038/nature10331 21822291 PMC3166416

[B42] Zhang H. Freudenreich C. H. (2007). An AT-rich sequence in human common fragile site FRA16D causes fork stalling and chromosome breakage in S. *Cerevisiae*. *Mol. Cell* 27 367–379. 10.1016/j.molcel.2007.06.012 17679088 PMC2144737

[B43] Zhu T. Wang L. Rimbert H. Rodriguez J. C. Deal K. R. De O. R. (2021). Optical maps refine the bread wheat *Triticum aestivum* cv. Chinese Spring genome assembly. *Plant J.* 107 303–314. 10.1111/tpj.15289 33893684 PMC8360199

[B44] Zou Y. Wan L. Luo J. Tang Z. Fu S. (2021). FISH landmarks reflecting meiotic recombination and structural alterations of chromosomes in wheat (*Triticum aestivum* L.). *BMC Plant Biol.* 21:167. 10.1186/s12870-021-02947-1 33823797 PMC8025513

