# Supplementary figures and images for: Variations of wheat (Triticum aestivum L.) chromosomes caused by the 5A chromosomes with complex cytological structure
Source: Front Plant Sci. 2022 Aug 29;13:992934. doi: 10.3389/fpls.2022.992934 (PMC9465395; doi:10.3389/fpls.2022.992934)

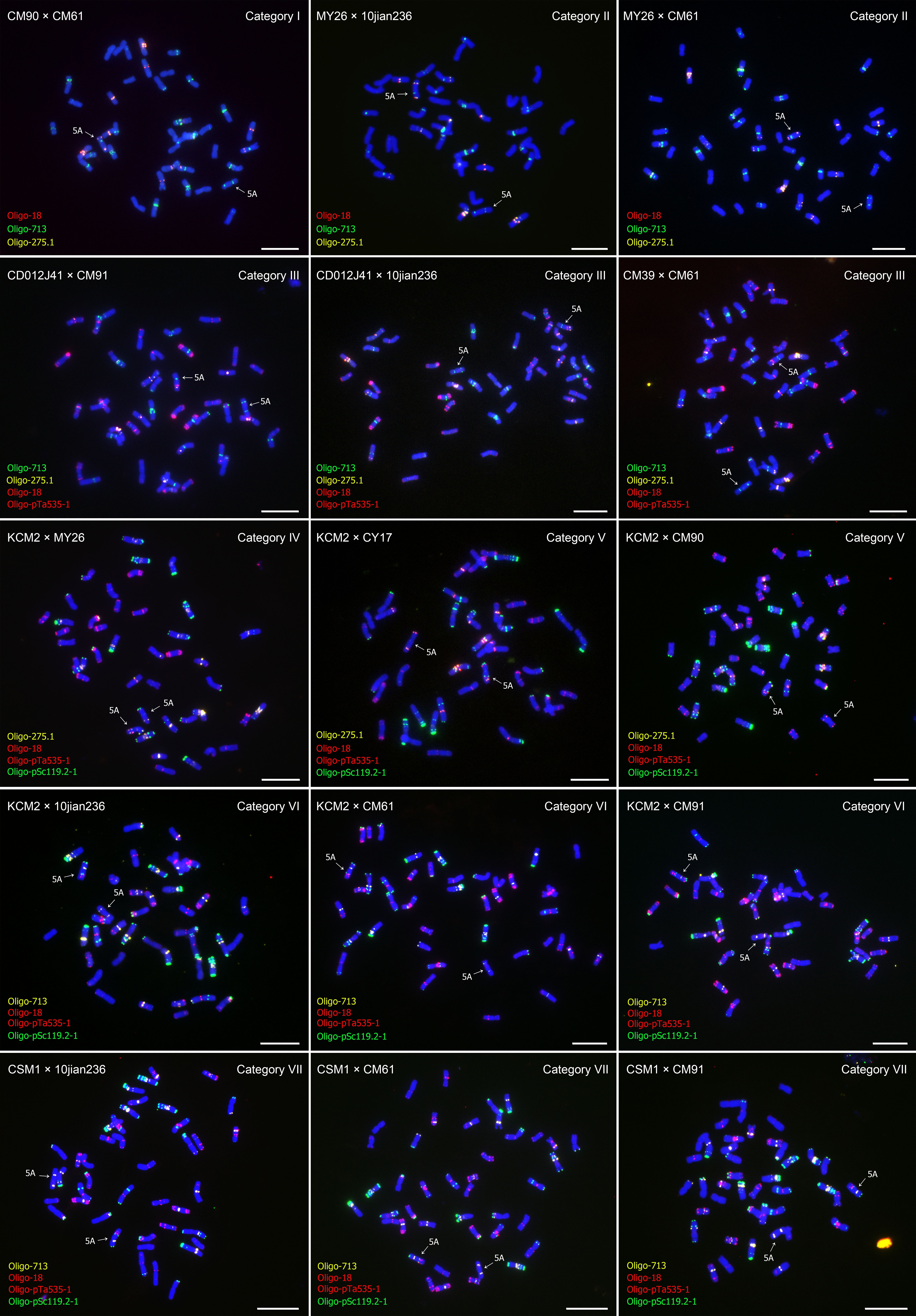

Supplement: Supplementary Figure 1 — Non-denaturing FISH (ND-FISH) analysis of the root-tip metaphase chromosomes of 15 F1 plants. The oligo probes Oligo-713 (green or yellow), Oligo-275.1 (yellow), Oligo-18 (red), Oligo-pTa535-1 (red), and Oligo-pSc119.2-1 (green) were used for ND-FISH analysis. The 15 F1 plants were divided into seven categories I, II, III, IV, V, VI, and VII. Scale bar, 20 μm. [file Image_1.JPEG]

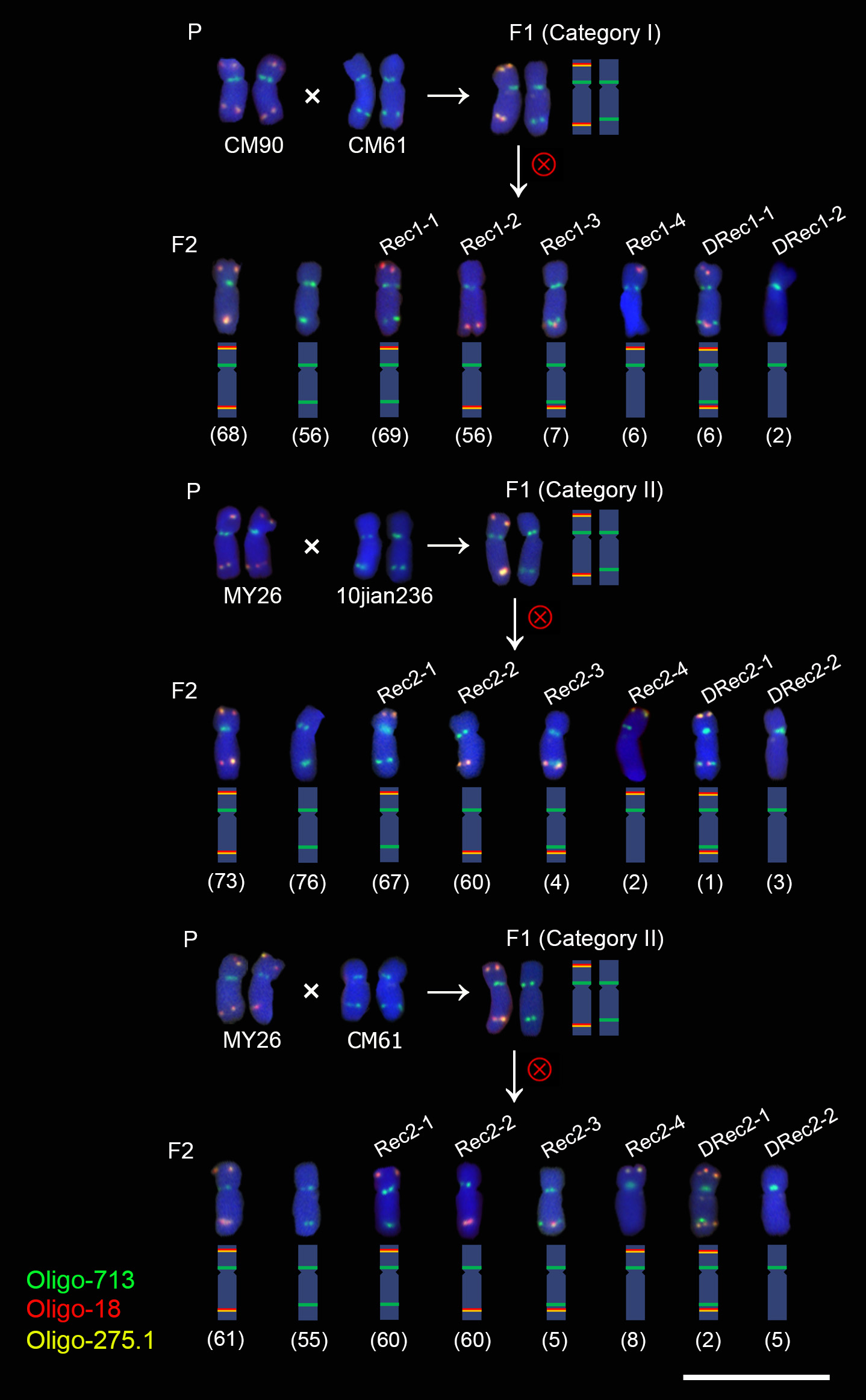

Supplement: Supplementary Figure 2 — Fluorescence in situ hybridization signal patterns of 5A chromosomes in the F2 plants from the I and II F1 categories. “P” indicates parental plants. “F1” indicates F1 generation. “F2” indicates F2 generation. Numbers in parentheses indicate the number of corresponding 5A chromosome. The schematic representation of each 5A chromosome is shown. Scale bar, 20 μm. [file Image_2.JPEG]

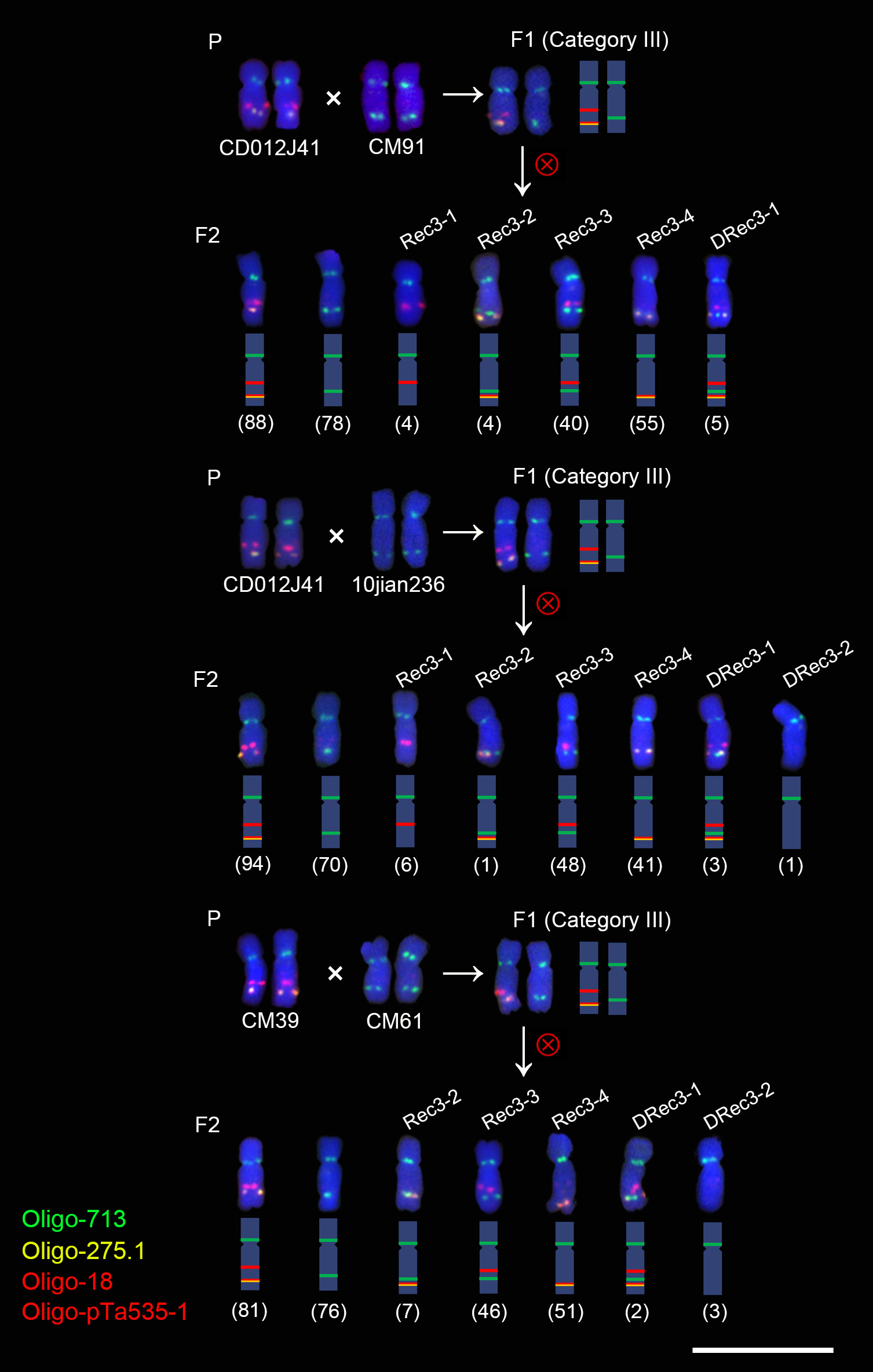

Supplement: Supplementary Figure 3 — Fluorescence in situ hybridization signal patterns of 5A chromosomes in the F2 plants from the III F1 category. “P” indicates parental plants. “F1” indicates F1 generation. “F2” indicates F2 generation. Numbers in parentheses indicates the number of corresponding 5A chromosome. The schematic representation of each 5A chromosome is shown. Scale bar, 20 μm. [file Image_3.JPEG]

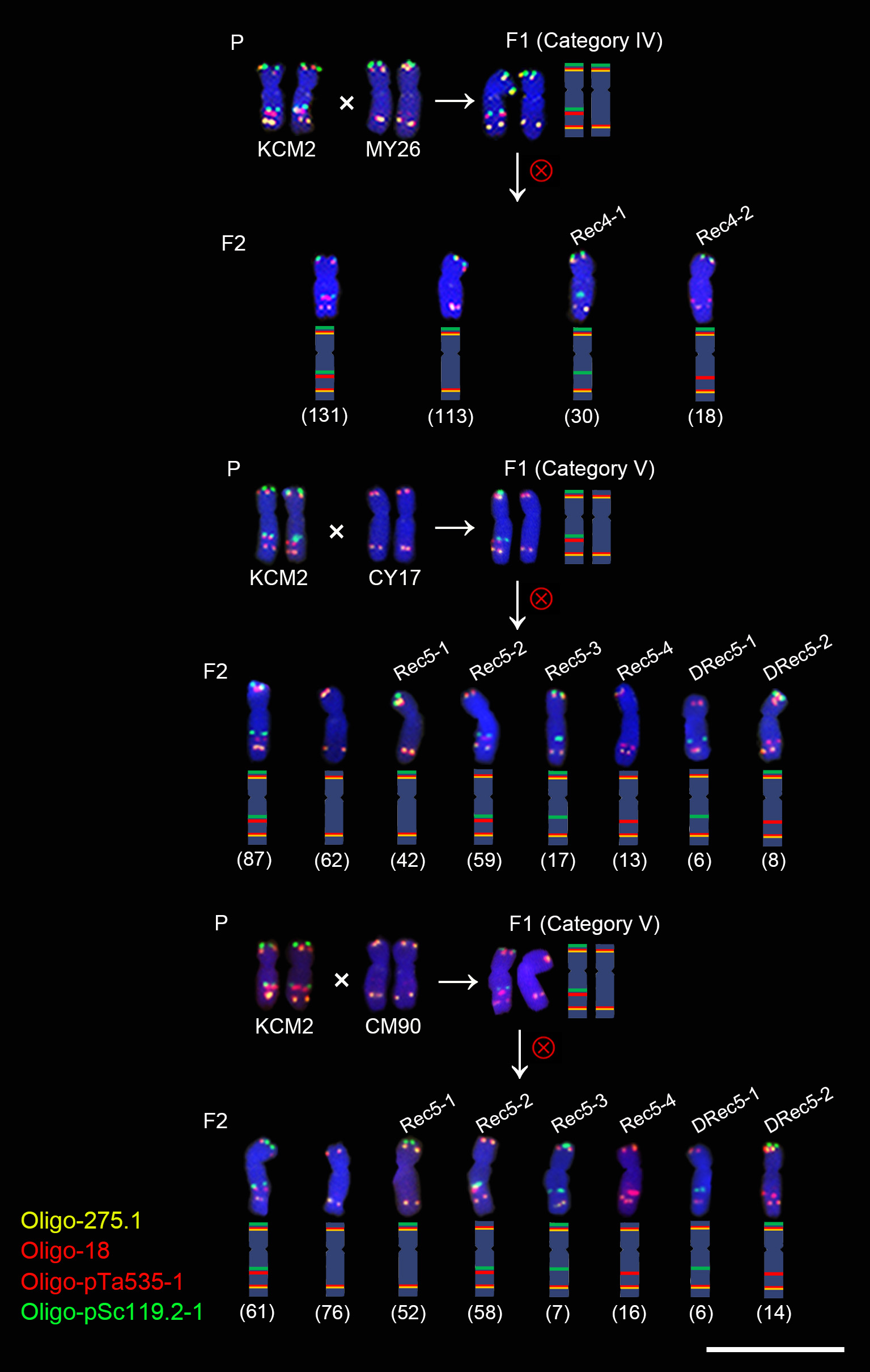

Supplement: Supplementary Figure 4 — Fluorescence in situ hybridization signal patterns of 5A chromosomes in the F2 plants from the IV and V F1 categories. “P” indicates parental plants. “F1” indicates F1 generation. “F2” indicates F2 generation. Numbers in parentheses indicates the number of corresponding 5A chromosome. The schematic representation of each 5A chromosome is shown. Scale bar, 20 μm. [file Image_4.JPEG]

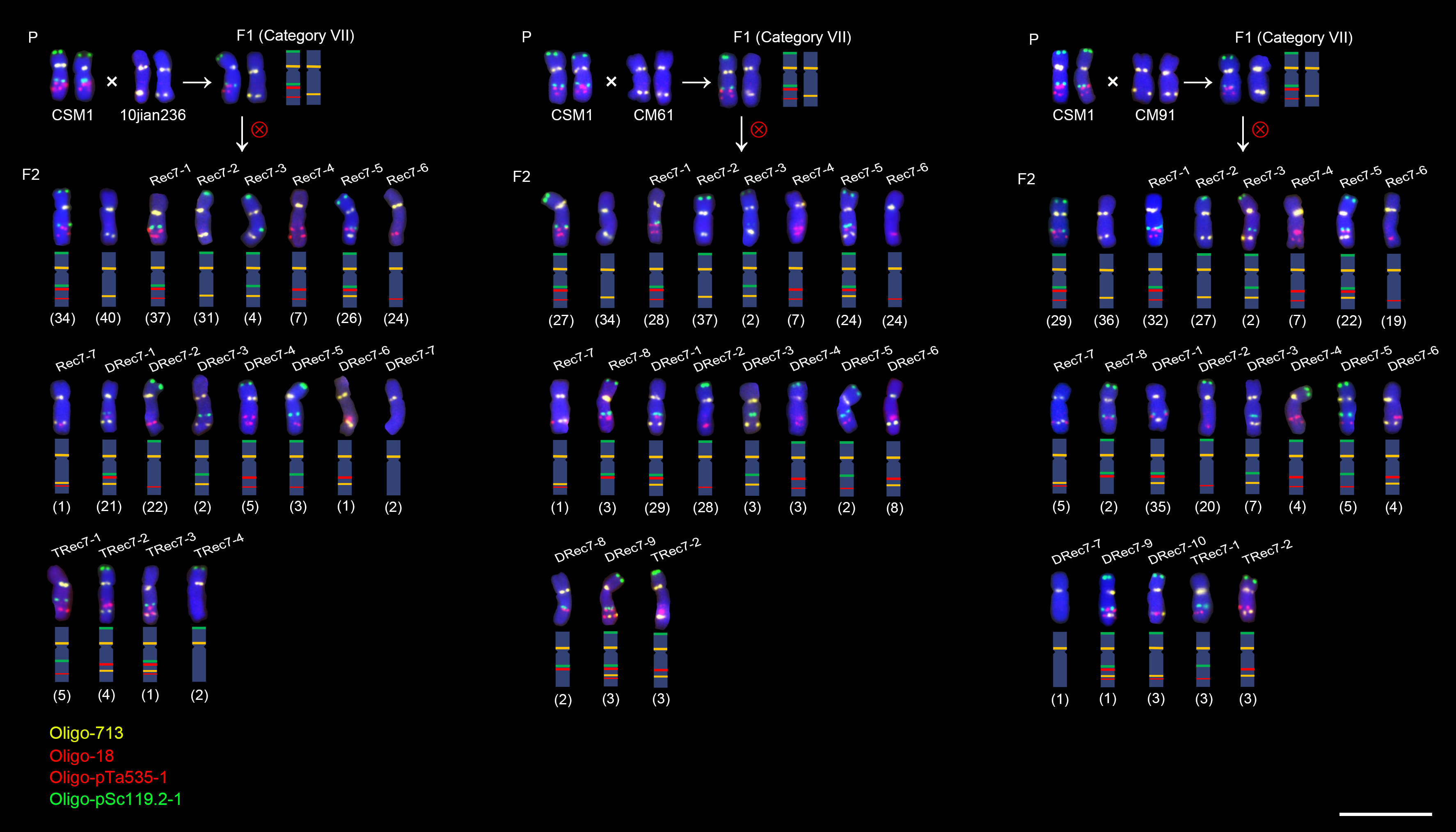

Supplement: Supplementary Figure 5 — Fluorescence in situ hybridization signal patterns of the 5A chromosomes in the F2 plants from the three hybrid combinations of category VII. “P” indicates parental plants. “F1” indicates F1 generation. “F2” indicates F2 generation. Rec7-n, DRec7-n, and TRec7-n represent the single recombination, double recombination and triple recombination, respectively. The numbers in parentheses indicate the number of each type of 5A chromosome. The schematic representation of each chromosome is shown. Scale bar, 20 μm. [file Image_5.JPEG]

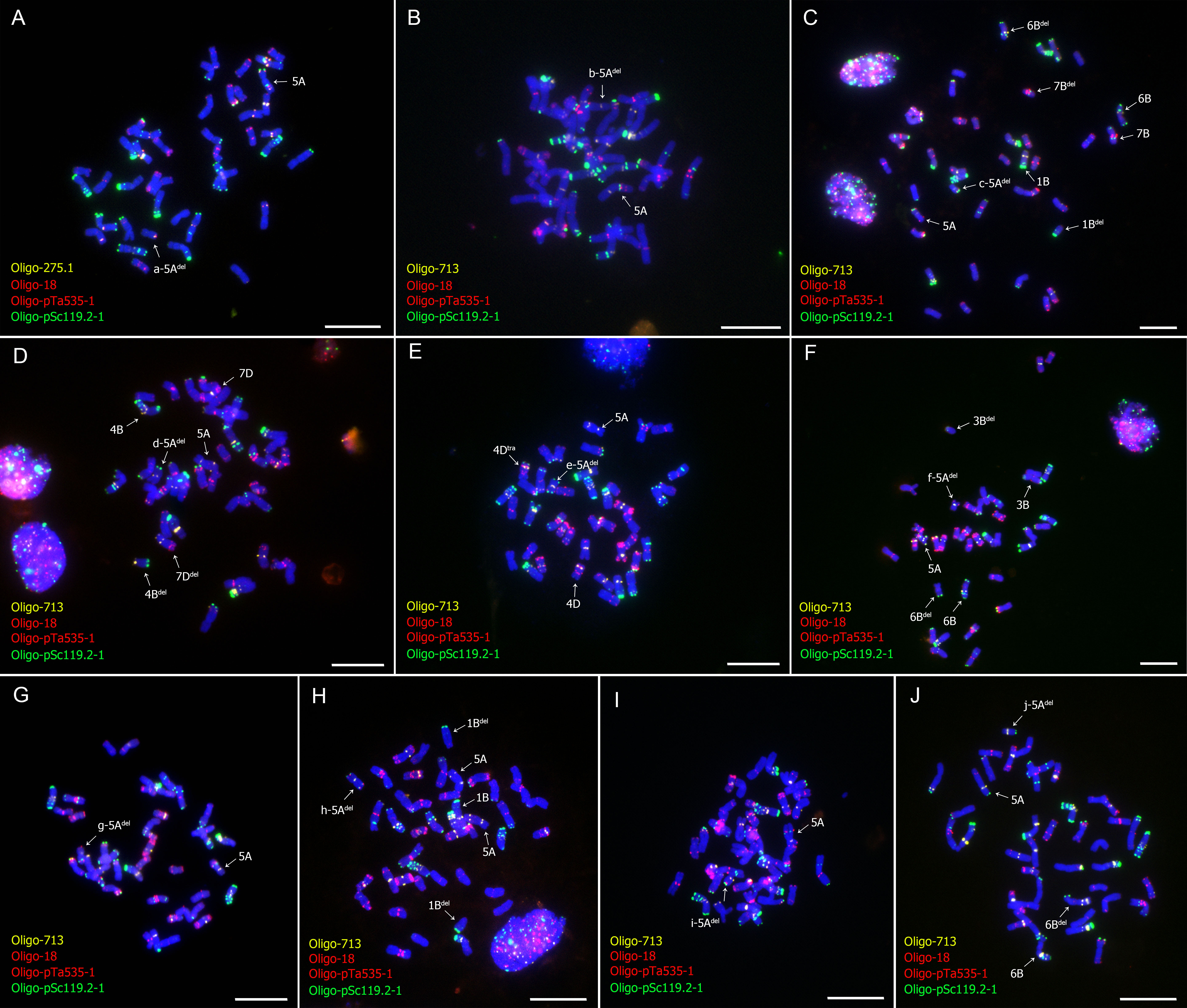

Supplement: Supplementary Figure 6 — Fluorescence in situ hybridization signal patterns of ten broken 5A chromosomes. The oligo probes Oligo-713 (yellow), Oligo-18 (red), Oligo-pTa535-1 (red), and Oligo-pSc119.2-1 (green) were used for ND-FISH analysis. Scale bar, 20 μm. [file Image_6.JPEG]

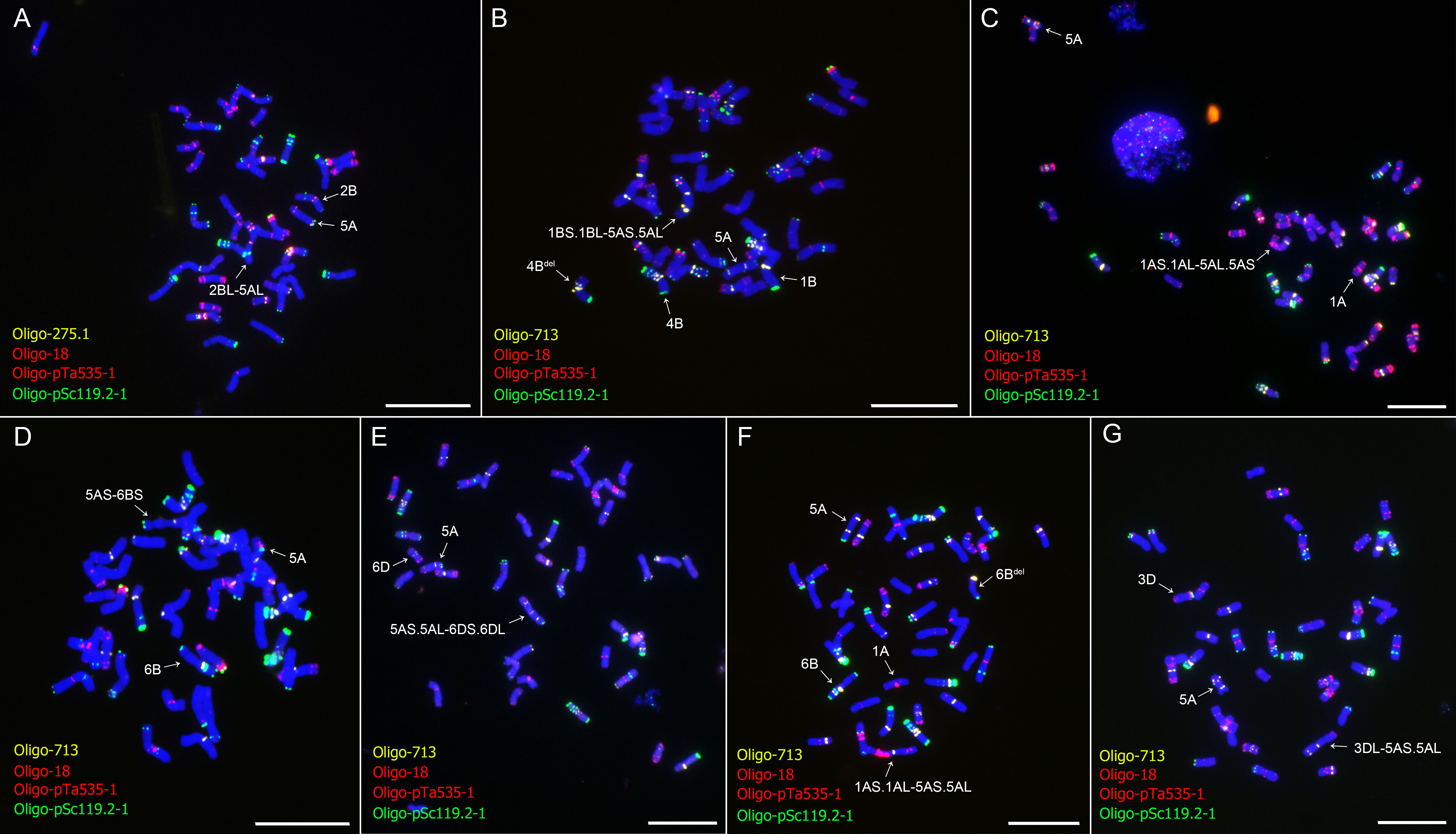

Supplement: Supplementary Figure 7 — Fluorescence in situ hybridization signal patterns of seven 5A non-homologous recombination chromosomes. The oligo probes Oligo-713 (yellow), Oligo-18 (red), Oligo-pTa535-1 (red), and Oligo-pSc119.2-1 (green) were used for ND-FISH analysis. Scale bar, 20 μm. [file Image_7.JPEG]
