# Supplementary material for: Variations of wheat (Triticum aestivum L.) chromosomes caused by the 5A chromosomes with complex cytological structure
Source: Front Plant Sci. 2022 Aug 29;13:992934. doi: 10.3389/fpls.2022.992934 (PMC9465395; doi:10.3389/fpls.2022.992934)
Supplement: Supplementary file 9 [file Table_2.DOCX]

**SUPPLEMENTARY TABLE 2** Frequency in breakage and non-homologous recombination of 5A chromosome

| Number | Hybrid combination | F_1_ category | The total number  of 5A in F_2_ ^*^ | Breakage | Non-homologous recombination |
| --- | --- | --- | --- | --- | --- |
|  |  |  |  | Number Frequency(%) | Number Frequency(%) |
| 1 | CM90 × CM61 | Ⅰ | 270 | 0 | 0 |
| 2 | MY26 × 10jian236 | Ⅱ | 286 | 0 | 0 |
| 3 | MY26 × CM61 | Ⅱ | 256 | 0 | 0 |
| 4 | CD012J41 × CM91 | Ⅲ | 274 | 0 | 0 |
| 5 | CD012J41 × 10jian236 | Ⅲ | 264 | 0 | 0 |
| 6 | CM39 × CM61 | Ⅲ | 266 | 0 | 0 |
| 7 | KCM2 × MY26 | Ⅳ | 292 | 0 | 0 |
| 8 | KCM2 × CY17 | Ⅴ | 294 | 0 | 0 |
| 9 | KCM2 × CM90 | Ⅴ | 292 | 1 0.34 | 1 0.34 |
| 10 | KCM2 × 10jian236 | Ⅵ | 278 | 4 1.44 | 1 0.36 |
| 11 | KCM2 × CM61 | Ⅵ | 285 | 3 1.05 | 3 1.05 |
| 12 | KCM2 × CM91 | Ⅵ | 270 | 1 0.37 | 0 |
| 13 | CSM1 × 10jian236 | Ⅶ | 274 | 1 0.36 | 1 0.36 |
| 14 | CSM1 × CM61 | Ⅶ | 268 | 0 | 0 |
| 15 | CSM1 × CM91 | Ⅶ | 268 | 0 | 1 0.37 |

* Including broken and non-homologous recombination of the 5A chromosome.
